# When National Origins Equal Socio-economic Background: The Effect of the Ethno-class Parental Background on the Education of Children Coming of Age in Switzerland

**DOI:** 10.1007/s12134-024-01129-w

**Published:** 2024-02-02

**Authors:** Eduardo Guichard, Milena Chimienti, Claudio Bolzman, Jean-Marie Le Goff

**Affiliations:** 1 LIVES Swiss Centre of Expertise in Life-Course Research, Lausanne, Switzerland; 2 University of Applied Sciences and Arts Western Switzerland, School of Social Work Geneva, HES-SO/HETS, Geneva, Switzerland; 3 University of Lausanne, Lausanne, Switzerland

**Keywords:** Migrants, Second generation, Education, Switzerland, Quantitative analysis

## Abstract

The educational outcomes of the descendants of migrants are important indicators of migrants’ incorporation into host societies and an indicator of intergenerational social im/mobility. This paper examines this relationship using data from a survey that follows a cohort of young adults, born between 1988 and 1997, who grew up in Switzerland. It looks at the relationship between the educational output of respondents and their parental migratory background, with the theoretical consideration that the family’s social capital is a starting point in the descendants’ trajectories. The paper is based on secondary data and exploratory cross-sectional quantitative analyses. The results highlight first a correspondence between migrant parents’ national origins and their socio-economic status—in other words, an ‘*ethno-class*’. Second, they show differences in educational outcomes between migrants’ descendants and native Swiss as well as between the migrants’ descendants themselves—which indicates a segmented incorporation process for both the first and the second generation, in confirmation of previous research. Third, results show that parental background and language region of residence are statistically significant in determining the level of education achieved by the migrants’ descendants, especially those with a low socio-economic status. Their social mobility is ‘limited’, and they remain mostly in vocational education. The paper concludes that the Swiss school system still fails to include the most unprivileged and that a glass ceiling remains for them.

## Introduction

Over the last century, Switzerland has known different waves of immigration with the result that the country’s population has more than doubled. Around a quarter of the population is nowadays of foreign origin (OFS, 2019) and has been settled in the country for many years, which has made their legal, economic, and social inclusion a key political concern since the 2000s (Fibbi & Wanner, 2009).

This concern is motivated by the assumption that migrants’ descendants remain more disadvantaged than the population of native parentage (Fibbi & Truong, 2015; Laganà et al., 2014), a claim that has inspired several studies aiming to explore the differences between migrants’ and natives’ descendants, particularly in the fields of education and access to the labour market. Earlier research has observed that migrants’ descendants (the so called ‘*second generation*’) have lower educational and occupational outcomes than those with native parentage (Park, 1928). Most research thus far has shown that the migratory background of the parents considerably affects the trajectory of their children (see Bolzman et al., 2003a, b for Switzerland; Portes et al., 2005 for the US; Schnell et al., 2015 for Europe). Portes and Zhou (1993) have theorised this phenomenon as ‘segmented assimilation’, showing both the transgenerational impact of the migratory background and the impact of exclusion from the host society.

Empirical investigations indicate that this relationship cannot be generalised to all national origins and that it varies according to the migrant family’s socio-economic status (SES) (Bauer & Riphahn, 2006; Gomensoro & Bolzman, 2016; Le Goff et al., 2023). Moreover, the long-term impact (seen through a life-course lens) of the family’s migratory background on the education and labour trajectory of their children leaves open questions. This generational influence of advantages or, disadvantages, highlights two possible trajectories: a ‘path-dependent’ relationship in intergenerational transmission, which assumes that parental resources and background play a capital role in their descendants’ lives, shaping their life-course trajectory; or a ‘cumulative-effect’ process which assumes that the different (and cumulative) choices, advantages or disadvantages, encountered across time can modify the trajectory and lead to different paths (DiPrete & Eirich, 2006; Kasinitz et al., 2008; Pudrovska & Anikputa, 2014).

This paper associates the notion of ‘segmented assimilation’ with the concept of ‘path dependence’. Hypothesising a ‘segmented-assimilation’ and therefore a ‘path-dependence’ model, this study explores the educational outcomes of the young descendants of migrants in Switzerland in relation to their family background through a cross-sectional approach using the LIVES COHORT Survey (LCS) dataset.^1^ This paper aims to answer to the following question: what is the effect of migrant’ parents background on the educational outcomes of their children?

## Theoretical Background and Previous Research

### Individual and Family Factors of Influence in Educational Outcomes: Path-Dependency Explicative Model

Former studies in migration have noted that migrants’ incorporation depends on the interaction between their individual and family characteristics and the institutional conditions that the receiving society offers and imposes on them, producing what the authors call a ‘segmented assimilation’ (Portes & Zhou, 1993; Portes & Fernández-Kelly, 2008; Rumbaut & Portes, 2001; Zhou, 1997), which manifests itself more clearly in generations subsequent to first-time migrants.

Earlier research reveals that a major determinant of migrant descendants’ educational outcomes is the parents’ pre-migration level of education, which represents a more important determinant than their occupational and economic position in the host society (Crul, 2013). When parents’ socioeconomic status (hereafter SES) is controlled for, migrant descendants seem to perform in a similar way to those with native parentage—and some even better (Crul et al., 2012; Le Goff et al., 2023; Portes & Fernández-Kelly, 2008; Rothon et al., 2009; Zhou & Kim, 2006). Sometimes the desire for upward social mobility (Kao & Tienda, 1995) and parental involvement (Fibbi & Truong, 2015; Schnell et al., 2015) can even compensate somewhat for the parents’ lack of educational capital.

Another determinant of migrant descendants’ educational outcomes is the place of birth, where the early socialisation of the young occurs and which can favour or decrease migrant descendants’ inclusion (Portes & Fernández-Kelly, 2008). Those born in Switzerland or arriving at around 5 years old—the age of entry into Swiss primary schools—seem to succeed better at school than those who arrived later (when parents’ SES is controlled for), given their access to and knowledge of the social environment, practices, and language of the host country from the start (Crul & Schneider, 2010). Bolzman et al. (2003a, b) found that the experience of migration during early childhood introduces disadvantages in school performance compared to those born in the host country.

Finally, performance at school varies according to gender: female migrant descendants perform better at school and have higher expectations than their male counterparts (see Alarcón et al., 2014 for Spain), especially among families with low SES. Women’s desire for emancipation and the pressure on young men for an early entry into the labour market both represent potential explanations for these gender differences among migrants’ descendants (Farris & de Jong, 2013). However, female migrant descendants’ final educational achievement is lower than for females with native parents when controlling for SES (for Denmark, the Netherlands, France and Italy, see Farris & de Jong, 2013; for Switzerland, Hupka & Stalder, 2004). These general results need to be disentangled between and within origins: for example, South American and Cuban women are more likely to reach tertiary levels of education than African-American natives or Mexicans in the USA, while Latina women study more diligently and obtain better employment than Latino men (Valdez & Tran, 2020).

Although these studies distance themselves from a linear impact of the parental migratory background on the educational trajectory of their children, they do see family and individual characteristics as key determinants. What remains unclear is the respective role of theses determinant (parents’ background, place of birth and gender) on the migrant descendants educational and professional trajectory. This paper examines the strength of parents’ background compared to other individual and structural determinants. Besides, few studies have analysed quantitatively the intergenerational reproduction of SES among migrants settled in Switzerland (Le Goff et al., 2023). This paper aims to show the influence of national origins on the intergenerational reproduction of the familial SES in Switzerland, explained by the congruence between certain nationalities among primo migrants and their low SES.

To explain the effect of the parental background on their children educational outcomes, we use the *path-dependency model* (Pudrovska & Anikputa, 2014). According to this model, resources associated with the parental background are transformed into educational capital, which is later converted into a socio-professional position. This means that the social position on the labour market depends on level of education attained which itself depends on the social position of the parents when the person is a child. However, there is not a direct effect of the social position of the parents during childhood; the educational system plays the role of mediator between parental background and the children’s socio-professional positions as we shall highlight in the next section.

### Institutional Factors of Influence on Educational Outcomes

Crul and Schneider (2010) added a new paradigm of analysis of migrant descendants’ trajectories, whereby schools play a more crucial role on educational outcomes than national integration policies. Educational models in Europe define the different institutional contexts ‘conditioning’ educational outcomes—such as performance and level of attainment (Crul, 2013). For instance, while migrant descendants in France are more likely to follow a general educational path leading to university, they are over-represented in the vocational track in Germany (Tucci et al., 2011) and Finland (Kilpi-Jakonen, 2014). Therefore, those in France are more likely to attain social mobility through higher-level qualifications than those in Germany (Tucci, 2015). As shown by (Griga & Hadjar, 2014), a ‘more stratified secondary educational system’ (with an early age selection) and ‘conservative welfare’ (Austria and West Germany) decreases the chances for descendants of migrants compared to those of native parents to attain a higher education degree and therefore the risk of ‘segmented assimilation’ is higher, whilst in low-stratified school systems, migratory background and low social position have less negative effects on the education trajectory.

This perspective is of particular importance in the Swiss context, as the country’s educational system is considered to be among the most unequal of all OECD countries, with high rates of SES reproduction between generations (Gomensoro & Bolzman, 2015) and important differences in educational outcomes between cantons and the national and SES origins of students (Felouzis & Charmillot, 2017; OECD, 2009). These differences are due:

Firstly, to an early and strong tracking into different performance-based groups that define, and often constrain, post-compulsory educational opportunities. Second, even if bridge-year courses exist, there are no easy paths to take because they entail a higher educational investment. Third, the early tracking often defines post-compulsory trajectories and reproduces SES at every level of these educational trajectories (Gomensoro & Bolzman, 2015, p. 76).

The early tracking system has two key moments. Around the age of 11 or 12 (depending on the canton), pupils are channelled to different tracks depending on their performance at primary school. This first selection is, however, not only based on the educational outcomes but also oriented by the teachers with more or less leverage according to the cantons of the students and their parents. A second key moment occurs at the upper-secondary level of education, which is divided between vocational education and general education. The split of the students between these two branches is mainly determined by the early selection occurring around the age of 12. In this sense, the educational orientation of the young occurs in Switzerland at an early stage of their life and often resulting from an institutional evaluation (Gomensoro & Bolzman, 2015).

In Switzerland, there are also regional differences: German-speaking regions offer fewer opportunities for educational advancement (to tertiary education) compared to French- and Italian-speaking regions (Felouzis & Charmillot, 2017). These differences can be explained by the late tracking in Italian-speaking regions which could improve equal opportunities and the low educational mobility in German-speaking cantons caused by the prevalence of the apprenticeship system (Bauer & Riphahn, 2006).

Independent of the region and origin, around 30% of all students in Switzerland finish compulsory education on a basic track, 39% follow an extended track (upper-secondary and vocational training at secondary level), and 28% remain on a Baccalaureate track (Gomensoro & Bolzman, 2015). Given these different determinants, it is hardly surprising that migrant descendants’ educational outcomes in Switzerland vary considerably, as shown below.

The study by Luthra, Soehl and Waldinger (2018: 924) based on US data innovates by disaggregating the mode of incorporation into its three components (policy reception, societal reception, and co-ethnic community) which allows to understand the respective roles of these dimensions compared to individual ones. They show a mobility between the first and the second generation but remain barriers for disadvantaged groups compared to those ‘more privileged immigrant groups and native whites (negative main effect on attainment)’.

This paper provides more evidence of the Swiss education system’s limits in including migrants and its paradox. On the one hand, the Swiss education system is often considered exemplary because of the low unemployment rates among young people (Garçon, 2016) and the important percentage of young people finishing their education with a degree. Indeed, the vocational training system offers access to study for a degree to those who did not want to choose or were unable to access a tertiary level of education and eases access to the labour market.^2^ On the other hand, it can be also criticised because of its high early-path selectivity, which reproduces the familiar baseline inequalities (Gomensoro & Bolzman, 2015, 2016). There exists, in particular, a glass ceiling for accessing the tertiary level (Falcon, 2016).

Whilst acknowledging that educational inequalities are structured by the stratification of the educational systems and embedded in a certain macro context of welfare and integration policies, this paper focuses on the individual and family determinants. It addresses the intergenerational reproduction of disadvantages through the lenses of the path dependence model and the ethno-class congruence in the parents’ background, claiming that migrant background and social origin are interrelated.

### Educational Outcomes of Migrant Descendants in Switzerland and the Ethno-class Effect

Previous research reveals an important gap in educational performance between descendants of migrants and Swiss natives (Fibbi & Wanner, 2009), as well as an important relationship between social inequalities, migratory origins, and educational outcomes (Gomensoro & Bolzman, 2016). The literature shows that upward educational mobility is generally less frequent among migrants’ descendants than among those with native parents; the former is also over-represented in non-traditional educational pathways oriented towards upward social mobility (for instance from a basic apprenticeship pathway to university studies). However, there are important differences depending on the parents’ national origins: whilst Bolzman et al. (2003a, b) observe that the descendants of Spanish and Italian natives perform better than Swiss natives with a similar socio-economic background, Laganà et al. (2014) show that immigrants from the former Yugoslavia, Turkey, and Portugal succeed less well than their Swiss counterparts.

Cases of intergenerational upward mobility among the descendants of migrants with low SES are explained, on the one hand, by their parents’ important investment in education and high aspirations (Geisen, 2014), which they see as offering their children socio-economic mobility. On the other hand, this educational mobility is explained by the school system in Switzerland, which offers several routes to qualification in cases of failure or drop-out—even if the selection of and orientation towards an academic track occurs earlier than in other countries (Fibbi & Truong, 2015; Gomensoro & Bolzman, 2015).

These studies also show that, in Switzerland, parents’ national origins are strongly related to household SES: this correspondence among primo migrants can mostly be explained by Switzerland’s labour recruitment policy, as was the case in other Central European countries (Wanner, 2004). To aid economic development after WWII, Switzerland massively recruited low-skilled workers (Mahnig, 2005; Piguet, 2013) to fill positions in the secondary labour market or domestic-oriented economy (Wanner et al., 2002). This recruitment was based on a guest-worker permit of stay that allowed foreign workers (then mostly originating from Italy and Spain) to stay for nine months in Switzerland and to return after three months for the same duration. Migrant stay was thought of as temporary, and they had restricted rights: for instance, they were allowed neither to bring their family nor to settle permanently in Switzerland. They were not, therefore, considered as citizens but only as temporary residents.

The relationship between certain nationalities and the low SES of the first-generation dates from this active labour recruitment in Switzerland’s neighbouring countries, where the unskilled and unprivileged saw an advantage to this emotionally and physically demanding migration, especially given the few rights to which they were entitled. The active recruitment of low-skilled labour migrants decreased in the 1970s after the xenophobic initiatives of the right-wing politician Schwarzenbach to limit the proportion of migrants to 10% of the population, despite the rejection of these initiatives by the population (Liebig et al., 2012). The recruitment also decreased because of the economic crisis in the mid-1970s. However, the correspondence between certain foreign nationalities and their SES continued with the migration of Portuguese, Turkish, and Albanian workers in the 1980s and with the arrival in Switzerland of refugees from Sri Lanka and the former Yugoslavia, especially those escaping from Kosovo, in the late 1980s and 1990s. Those who went to Switzerland were mostly from rural regions and low-skilled, unlike the Tamil Sri Lankans who went to the UK (Moret et al., 2007) and the Kosovar population (Burri Sharani et al., 2007). The consequence of this panorama is that primo migrants in Switzerland were usually offered low-skilled positions on the local labour market. Such inclusion defines the family’s financial capital (Bauer & Riphahn, 2006) and development of social networks, both of which represent social capital that may have increased the possibilities for children of immigrant families (Dahinden, 2005; Levitt, 2009).

This period of active foreign-labour recruitment is also characterised by an absence of national integration policies for migrants until the 2000s, which means that insertion in the labour market represented their main pathway to inclusion in the host society—as well as representing a risk of social reproduction (Cattacin & Chimienti, 2006; Chimienti et al., 2021a).

The correspondence between some primo-migrant nationalities and their low SES creates what is called in this paper an *ethno-class*, drawing on Portes and his colleagues (Portes & Zhou, 1993; Rumbaut & Portes, 2001), who defined it as the social position occupied by migrants in the socioeconomic structure of a society according to their national origins. This notion emphasises that, even if the social structure allows for a certain social mobility, the social position of the parents is transmitted in relative terms to their descendants, in particular through the educational system. In other words, this paper argues that the existence of an *ethno-class* in Switzerland puts up a specific barrier against the socio-economic mobility of the next generation(s). This congruency led to the unfounded perception relayed in the Swiss media of migrants of these origins as uneducated manual workers and to a false assumption about the impact of nationality on the educational trajectory of the second generation (Hoffmann-Nowotny, 1985).

### Hypothesis

This study hypothesises that a ‘*path-dependency*’ *model* governs the educational outcomes of migrants’ descendants. This model (Pudrovska & Anikputa, 2014) assumes that aspects such as parental resources and family background play a capital role in the educational trajectory of migrant children. Thus, this analysis focuses on the SES and nationality of the parents, expecting that their offspring’s varying starts in life will probably also differ from the educational outcomes of Swiss natives. Resources related to the parental socio-cultural background are converted into children’s educational resources. These resources could even be valued, if children’s level of education becomes higher than the parental ones or, alternatively, could be devalued if it becomes lower (Levy & Bühlmann, 2016). According to this hypothesis, there is however a mediating effect of the education level reached by the children.

Second, this paper assumes that migrant background and social origin are interrelated and that this ethno-class characteristic creates a specific drag on the social mobility of the next generation. This additional barrier cannot be redressed by the school system, which is also very selective, and explains segmented assimilation among migrant descendant.

## Data and Methods

This paper is based on secondary data from the LIVES COHORT Survey (LCS), an annual longitudinal survey following a cohort of young adults, born between 1988 and 1997, who grew up in Switzerland (Spini et al., 2019). The LCS study is an original research designed and collected by the Swiss National Centre of Competence in Research (NCCR) Overcoming vulnerability: Life course perspective [LIVES Centre] and the Swiss centre of expertise in social sciences (FORS) which focuses on migrants’ descendants. Our research team was part of the NCCR LIVES and participated to the data collection and then commissioned to analyse the LCS database with the following main aim:

to describe the life paths to adulthood in Switzerland today and to compare young adults from the second generation to those whose parents have grown up in Switzerland (either born there or arriving as minors) [and to compare] the life trajectories of children of migrants with those of Swiss natives (Spini et al., 2019, p. 400).

To enable this comparison, youth from the second generation were over-represented in the LCS in contrast to other Swiss (longitudinal) surveys (Chimienti et al., 2021b; Le Goff et al., 2023). The LCS focuses in particular on the descendants of economic migrants from Southern and Eastern Europe who characterise the main migration flows into Switzerland in recent decades. Ex-Yugoslavia and Turkey nationalities are therefore purposely over-represented in the sample^3^ (Spini et al., 2019). As a result, our sample consists (apart from Swiss nationals) mainly of the descendants of migrants from Portugal (16%) and ex-Yugoslavian countries (14%), with a smaller number of Italian and Spanish descendants (7%) or Turks (4%) (Table 1).

**Table 1:** Descriptive statistics sample

Variable	*n*	Percentage
Sex		
Women	480	51%
Men	448	48%
* Missing*	6	1%
Age		
21–24	556	60%
25–32	377	40%
*Missing*	1	0%
Education ego		
Compulsory	57	6%
Vocational training	482	52%
Upper secondary	160	17%
Tertiary	235	25%
Parents nationality		
Switzerland	269	29%
Portugal	145	16%
Ex-Yugoslavia^a^	130	14%
Switzerland+	121	13%
Kosovo	99	11%
Italy, Spain	61	7%
Turkey	40	4%
Europe+	25	3%
Europe^b^	23	3%
*Missing*	21	2%
Language region		
German	441	47%
French	351	38%
Italian	61	7%
Bilingual	81	9%
Total sample	934	

^a^Ex-Yugoslavia = Serbia, Croatia, Bosnia, Macedonia, Albania, Montenegro

^b^Europe = Austria, Belgium, Czech Republic, France, Germany, Ireland, Italy, Norway, Poland, Portugal, Romania, Sweden, the UK

This research design has two main consequences: first, the sample is strongly concentrated on the descendants of migrants with European origins, especially from ex-Yugoslavian countries and Portugal. This concentration in the LCS survey provides a clear image of migration in Switzerland in a specific period—those young people whose parents migrated between 1980 and 1990 as, mostly, labour migrants from Southern Europe^4^ and refugees from former Yugoslavian countries and Turkey (Afonso, 2004). The focus of LCS on ‘disadvantaged migrants’ allows more accurate analysis within this group, as those from Northern Europe and the Americas represent a minority among migrants.

Although the LCS sample is about the descendants of migrants, it also includes information about their parents and also about politically displaced migrants who are supposed to have greater socio-economic diversity than economic migrants, who correspond to the manual workers recruited during the 1960s to 1980s.

This article uses LCS data from Waves 1 (collected in 2013–2014) to 6 (collected in 2018–2019)—a total of 1962 cases. The analysis focuses, however, on those in the sample who responded to the ‘Social Origins’ (SO) module of the questionnaire in the third wave (990 cases). From this sample, 56 cases from other nationalities were excluded in order to improve our results on the main national groups studied. Therefore, the sample used in the analysis for this paper is composed of 934 cases of young people between 21 and 32 years old (last participation in the survey) who grew up in Switzerland; descriptive statistics about their gender, age, parental nationality, and language region are provided in Table 1.

To explore the link between parental background and the level of higher education achieved, this study begins by examining the differences in the parental backgrounds of our respondents, looking at their national and socio-economic origins and combining their SES and nationality to build a typology. Three types of analysis are used in this paper: *Multiple Correspondence Analysis* (*MCA*), *Hierarchical Classification on Principal Components* (*HCPC*), and *Multinomial Logistic Regression* (*MLR*).

In order to develop a parental-background typology based on the relationship between parental nationality, educational level, and occupation, an MCA followed by an HCPC was used. An MCA is a statistical exploratory multidimensional method that allows examination of the associations between categories of variables (Lebart et al., 2000). The advantage of this analysis is that it enables the simultaneous examination of the different variables and their categories, the association between which is interpreted by statistical measures based on a spatial representation of distance and proximity between the latter (Lebart et al., 2000).

The distances obtained with the MCA were used in the HCPC to develop a typology of parental background. The HCPC is an innovative process that allows the creation of a classification (type) through an automatized process based on the results of an MCA (Husson et al., 2010). The HCPC groups and classifies cases according to the distance (and proximity) between their characteristics (variables’ categories) obtained in the MCA (Lebart et al., 2000). This procedure allows us to obtain different ‘types’ of parental background based on the main socio-economic characteristics of cases’ parents. This strategy of analysis of combining MCA and HCPC allowed us to build a typology of the parental background of the young people in our sample, while representing statistically the social stratification of the migration produced by the evolution of the migratory policies in Switzerland. In addition, the MCA analyses allow us to give equal weight to different characteristics of fathers and mothers in representing the family’s SES. The spatial representation of the MCA becomes a representation of the “social space” (Cayouette-Remblière & Ichou, 2019; Savage et al., 2015), and the use of the HCPC makes it possible to represent different types—configurations of family SES by an “optimal” division of the classes based on interpretability criteria (Husson et al., 2010). With this first combined analysis, we propose an inductive categorisation based on the relation between the variables as they emerge from the processed data, while using a cumulative approach of other categorisations such as the ESEC. In a nutshell, we propose an exploratory approach for the categorisation of the parental SES, based on a complementarity of methods in the reduction of information on the origins of the young descendants of migrants.

In a second stage of the analysis, we examined the relationship between the individual characteristics of young migrant descendants and their educational output expressed as the highest level of education attained by them in their last participation in the LCS. With these analyses, this study expects to investigate the influence of the parental background compared to the individual characteristics (gender and birthplace) of migrants’ young descendants and the language of their region of residence on their educational outcomes. In this stage of the analysis, a reduced sample is used that considers only the individuals who were assumed to have already finished their education—i.e. those between 25 and 31 years old.

Thirdly the MLR analysis allows us to predict to which type of education the children will belong according to the different individual characteristics (Petrucci, 2009). The MLR model considers as a dependent variable the highest level of education reached by the young^5^—at the moment of the last interview—in relation to the parental background typology obtained in the first stage of our analysis, the gender, the birthplace (born in Switzerland or abroad), and the region’s language. The final goal of MLR analysis was to examine the influence of the (parental) background and individual sociodemographic characteristics in determining the educational level achieved by the migrants’ descendants.

In order to perform these different analyses, a set of constructed variables were developed to deal with different methodological issues:

- *Nationality of the parents:* Although, in most cases, the nationality of both parents was registered, our analysis considered only the nationality of one parent in order to avoid putting too much weight on it, which would have blurred our results. It was possible to count only one nationality given the important national homogamy among the parents: 85% of the sample had the same nationality and only 2% were binational couples, in which case only the father’s nationality was considered (Table 1). The distinction between mixed couples—Swiss with a non-European partner and Europeans with a non-European partner—was retained because of their higher frequency in the sample (16%).
- *Mother’s and father’s level of education:* The highest level of education reached and declared by the mother and by the father were included in the analysis as separate variables.
- *Mother’s and father’s occupation:* These variables consider the respective employment of the parents when their child was 15 years old in order to examine the inclusion of the parents in the labour market after their arrival in Switzerland. For classifying parental occupation, the LCS used a shortened version of the European Socioeconomic Categories (ESeC). Anchored in a neo-Weberian tradition, this classification is built on employment relations, classifying professions according to the status ‘employed vs. independent’ and the level of skills (Tillmann, 2020). Establishing nine main classes, this classification allows us to grasp nuanced forms of professional mobility (Brousse et al., 2007).^6^

For this analysis, we developed a simplified version of the ESeC that considers only five occupational categories—the social positions of the occupation in which most primo migrants work in the Swiss labour market (see Table 2)—taking into consideration the relationships of dependence at work such as (i) ownership (vs. employment); (ii) the degree of relation to authority (i.e. manager vs. executor); and (iii) the degree of skills required for occupying a position in the labour market.

**Table 2:** The European socio-economic classification of occupations: original and recoding used

ESEC original	Relation employment/qualification	ESEC recode
Large employers, higher managers/professionals	Service relationship qualified	(i) Manager/liberal employee
Lower managers/professionals, higher supervisory/technicians	Service relationship qualified	(i) Manager/liberal employee
Intermediate occupations	Labour contract qualified	(ii) Intermediate employee
Small employers and self-employed (non-agriculture)	*Petite bourgeoisie*	(iii) Small/self-employers
Small employers and self-employed (agriculture)	*Petite bourgeoisie*	(iii) Small/self-employers
Lower supervisors and technicians	Labour contract qualified	(ii) Intermediate employee
Lower sales and service	Labour contract sector tertiary	(iv) Lower-grade white-collar workers
Lower technical	Labour contract not qualified	(v) Skilled, semi- and non-skilled workers
Routine	Labour contract not qualified	(v) Skilled, semi- and non-skilled workers

Five types of occupational classification were obtained:

i) *Manager/liberal employee* corresponds to large employers or superior qualified workers/liberal professionals in a contractual relationship of services with the employer.
ii) *Intermediate employee* refers to intermediate qualified workers with a labour contract.
iii) *Small and self-employed* are the self-employed owners of small enterprises.
iv) *Lower-grade white collar* represents contract workers in sales and service sectors.
v) *Skilled, semi-skilled, and non-skilled* are workers in the lower positions of the labour market with the most vulnerable service contracts. Here, the dominant element is their low position in the labour market compared to the other categories (Table 2).

## Results

In this section, the typology of parental background of the migrants’ descendants and its use is described. The typology sheds light on the relationship between the national origins of migrant parents (primo migrants) and SES. In a second stage, we include the typology of parental background with the birthplace, gender, and language of the region of residence of the young in a regression model (MLR). This analysis shows that parental background is statistically significant in predicting the educational level achieved by the young—in particular those with low-SES parents.

### Typology of Parental Background

**Fig. 1:**
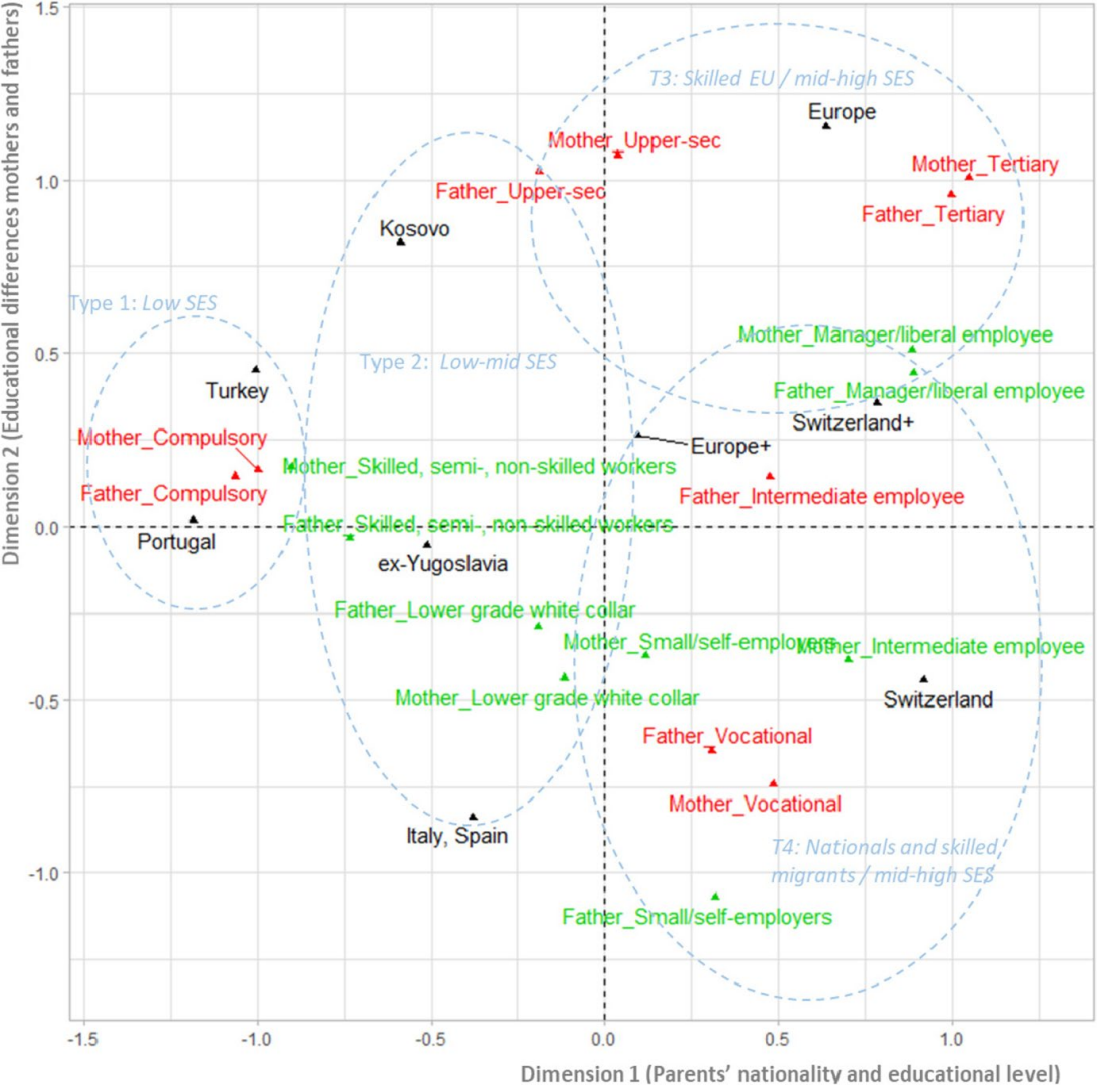
Relationship between parents’ nationality, education, and occupation in multiple correspondence analyses. Ex-Yugoslavia = Serbia, Croatia, Bosnia, Macedonia, Albania, Montenegro. Europe = Austria, Belgium, Czech Republic, France, Germany, Ireland, Italy, Norway, Poland, Portugal, Romania, Sweden, the UK

Performing *Multiple Correspondence Analysis* (MCA) and a *Hierarchical Classification on Principal Components* (HCPC) resulted in an initial typology of seven types of background based on the nationality, education, and occupation of the parents. This typology represents, synthetically, the socio-economic position of primo migrants already established in Switzerland. Although the MCA analysis shows that there is a high degree of difference in the association between variables considered in the construction of the background typology (the first two dimensions retain 21% of the variability), it is still adequate to validate our results. Figure 1 presents the spatial representation of the associations between the different parental national origins, levels of education and occupation. Results of the MCA show that cases were mainly grouped firstly according to the relation between the parents’ nationality and the mother’s education and occupation (Dimension 1, eta square is 0.68 for nationality, 0.64 for mother’s education, and 0.6 for mother’s education) and secondly according to the educational differences between the mothers and the fathers (Dimension 2, eta square education of the mother is 0.46 and 0.4 for the father).

The results of the ACM suggest the existence of an ‘ethno-class’ structure of migration in Switzerland (as SES is related to nationality) which can be explained by the principle of guestworker recruitment, as highlighted in our introduction. This confirms previous research that stated that migrants arriving at that time from South-Western and South-Eastern Europe were characterised by lower levels of education than migrants from Central Europe (Peccoraro, 2011; Wanner et al., 2002). Whilst the majority of Western-European countries experienced a similar structure of migration in the 1980s and 1990s, the national origins of migrant populations differ by country (Bailly et al., 2004; Tucci, 2015). For instance, Lessard-Phillips and Ross (2012) observe that the educational gap between migrants from the former Yugoslavia (higher levels) and Turkish citizens is bigger in Germany than in Switzerland. This configuration of their education between these Turkish and ex-Yugoslavian migrant parents is also noticeable in our ACM results.

Another aspect that supports this ‘ethno-class’ relationship concerns the educational levels within each nationality and between mothers and fathers, given the strong educational homogamy among spouses (Ravazzini et al., 2017). Figure 1 shows that parents’ vocational training is the most common level of education among Swiss nationals and primo migrants from Italy and Spain. (Parent) spouses from these origins show educational heterogeneity. Mixed European and mixed-Swiss couples attained mostly a tertiary level of education. In contrast, parents from Portugal and Turkey show low level of education, while those from former Yugoslavian countries, Italy, and Spain have low to high levels of education with a high heterogeneity within each group of nationality.

In the second stage of our analysis, the *Hierarchical Classification on Principal Components* (*HCPC*) allowed us to build a typology of parental background based on the results of the MCA on the relationship between parents’ nationality, level of education, and occupation in the Swiss labour market. The classification obtained with the HCPC splits the primo migrants into four types or profiles of parental background.

This analysis allowed us to automatically assign each case to a determined type or profile depending on the categories of nationality, education, and occupation that characterise the type. This typology was later compared with the distribution of the parents’ nationality (Table 3) in order to test the adjustment of our typology to the data. These types are delimited by dashed circles in Fig. 1, thus highlighting the associations between parents’ nationality, education, and occupation. The association between these variables is also proved by the *p*-value of the parents’ nationality (< 0.01) associated with the clusters obtained in the chi-square test.

**Table 3:** Typology of parental background by nationality (N valid) (%)

Type parental background	Nationality of parents									% type in N
	PT	TR	ex-Yug (^a^)	KO	IT, ES	EU (^b^)	EU+	CH	CH+	
T1: Low SES	98	88	–	–	–	–	–	–	1	20
T2: Low-mid SES	1	3	90	89	80	–	40	3	6	31
T3: Skill EU/mid-high SES	–	–	–	–	–	100	–	–	–	3
T4: Nationals and skilled migrants/mid-high SES	1	10	10	11	20	–	60	97	93	47
N origins	145	40	130	99	61	23	25	269	121	913

*CH* Switzerland, *PT* Portugal, *IT* Italy, *ES* Spain, *KO* Kosovo, *TR* Turkey, *ex-Yug* ex-Yugoslavia, *EU* Europe, *CH*+ = Swiss with non-EU partner, *EU*+ European with non-EU partner

^a^Ex-Yugoslavia = Serbia, Croatia, Bosnia, Macedonia, Albania, Montenegro

^b^Europe = Austria, Belgium, Czech Republic, France, Germany, Ireland, Italy, Norway, Poland, Portugal, Romania, Sweden, the UK

Parental background Type 1, which represents 20% of the cases in our sample, is characterised by migrant parents occupying lesser-qualified positions in the Swiss labour market (as skilled, semi- and non-skilled workers) and showing mainly low educational capital. This type includes mainly migrants from Portugal and Turkey. In this paper, we name this group ‘Low SES’. Virtually, all (98%) Portuguese and 88% of Turkish parents in our sample match this profile (Table 3).

Parental background Type 2, which represents 31% of our sample, regroups migrants from ex-Yugoslavian countries, Kosovo, Italy, and Spain who have low to mid-levels of education but who, at the same time, seem to occupy heterogeneous positions in the labour market. This group is completed by 40% of European couples with a non-EU partner. This group was labelled as ‘Low-mid SES’.

Type 3 regroups a small part of the sample (3%) composed exclusively of couples of European origins with mid to high levels of education and insertion in the Swiss labour market. This profile coincides with the increase in highly qualified migration from Austria, Germany, France, and the UK since the 2000s (Wanner & Steiner, 2018). This group was labelled ‘Skilled EU-mid to high SES’.

Type 4, which is the largest (44%), assembles people with medium to high levels of education occupying medium to high positions in the labour market as well as self-employed people. Almost the whole sample of native-Swiss and bi-national (Swiss and another nationality) couples constitute this higher SES type of background. This group also includes 60% of European couples with a non-EU partner, as well as 20% of couples from Italy and Spain. Interestingly, 10% of migrant couples from Turkey, Kosovo, and ex-Yougoslavian countries were identified as having a better position than their compatriots. This fourth group was labelled ‘Nationals and skilled migrants-mid to high SES’.

This means that some primo migrants escape from the *‘ethno-class’* relationship identified for the majority because of their higher SES before migration. For instance, those coming from the former republic of Yugoslavia benefitted from a long-term education system, supported during Tito’s communist regime, which tended to equalise potential social and economic differences (Spini et al., 2014). Such an educational background might have allowed some respondents to find skilled positions in the Swiss labour market although the majority of ex-Yugoslavians in our sample have a low social and economic background. This result can also be due to a methodological limitation of the ESeC classification, which systematically interprets self-employment as a higher category of occupation than those who are merely employed (Brousse et al., 2007), despite the fact that some self-employment can be very precarious.

In addition, this typology shows the congruence between nationality and SES among migrants arriving in Switzerland in the 1980s and 1990s due to the country’s labour and migration policy, which caused a segmented incorporation of migrants in Swiss society.

### The Relationship Between Background and Level of Education Attained by Migrants’ Descendants

To analyse the relationship between parents’ background and their children’s level of education, we performed a multinomial logistic regression (MLR), using the highest level of education attained by the young as the dependent variable and controlling for gender and place of birth and language of young descendants. This MLR includes only the ‘older’ individuals in our sample who, in theory, had finished their education—i.e. those between 25 and 31 years old (*n* = 370).

For our analysis, the vocational level of education was considered as the reference category for the model, because it is the most usual type of education attained by students in Switzerland and by the youth in our sample. In addition, the Type 4 parental background is used as the category of reference, composed of men who were born in Switzerland to native Swiss parents, with higher education and a higher labour-market position. This type of profile was used as we assumed that taking as a reference the more standard privileged cases would help to describe the less-privileged, due to the important contrast in their situation compared to those who face higher barriers in social stratification (assumed to be born abroad and less-qualified women).

The results of the MLR show that young people whose parents are less privileged (those categorised as Type 1 and 2 who have low education and low positions on the Swiss labour market and who happen to be of Portuguese, Turkish and, to a lesser extent, former-Yougoslavian, Kosovar, Italian, and Spanish origins) are much less likely to achieve an upper-secondary (−1.2**, see Table 4) compared to a vocational-level. Those in the Type 1 (−1.4***) are a little less likely to achieve tertiary education than those in the background Type 2 (−1.2***, see Table 4).

**Table 4:** Educational level attained as predicted by parental background, birthplace, and gender—MLR coefficients and standard errors (§)

Ref: vocational	Educational level attained by migrants’ descendants								
	Compulsory			Upper-secondary			Tertiary		
	*B*	Exp(*B*)	S.E	*B*	Exp(*B*)	S.E.	*B*	Exp(*B*)	S.E
*Constant*	−4.559		1.155	−2.138		0.478	0.157		0.236
T1: *low SES*	−0.795	0.451	0.821	−1.237**	**0.29**	**0.554**	−1.463***	**0.231**	**0.336**
T2: *low-mid SES*	−0.415	0.66	0.765	−1.255**	**0.285**	**0.527**	−1.247***	**0.287**	**0.296**
*Ref: T4 ch-mid-high SES*									
Not born CH	1.086*	**2.962**	**0.658**	0.792	2.209	0.469	−0.096	0.908	0.298
*Ref: Born CH*									
Woman	−0.679	0.507	0.647	−0.033	0.968	0.417	−0.015	0.985	0.241
*Ref: man*									
French	3.126***	**22.788**	**1.083**	1.678***	**5.357**	**0.471**	0.817***	2.263	0.271
Italian	2.003	7.41	1.454	0.031	1.032	1.111	0.424	1.529	0.484
Bilingual	2.253	9.515	1.477	0.046	1.047	1.117	0.668	1.951	0.448
Ref: German									

Note: *p < 0.10, **p < 0.05, ***p < 0.01

(§) Background type 3 was not considered in the MLR because the low number of cases after filtering by age group (*n* = 4)

Model fit: Cox et Snell (.017), Nagelkerke (.203)

Significant variables in the model are highlighted in bold

Level of significance is indicated by the asterisks

This difference in the results of both types highlights the educational and occupational diversity among migrants from ex-Yugoslavian countries, as well as an evolution of SES levels among migrants from Italy and Spain compared to the waves of Italian and Spanish working migrants of the 1950s in Switzerland. This result confirms that the vocational level is the most common degree of education for the children of migrants who come from countries considered as workforce providers or who arrived in later forced migration waves (such as members of the Turkish and Balkan diasporas) while, at the other extreme, young people with a low SES parental background are also less likely to attain a tertiary education compared to Swiss nationals.

The importance of the early socialisation of migrants is also highlighted by the relation between the *birthplace* and the *level of education.* Migrants’ descendants who were not born in Switzerland are more likely to achieve only a compulsory level of education rather than a vocational level. The descendants of migrants who were *not* born in the host society tend to drop out of school earlier. In contrast, those who were born in the host country seem to be more protected from the risk of dropping out of the school system before gaining a degree.

Finally, the results show that young migrant descendants living in French-speaking cantons are significantly more likely to reach all educational levels rather than only the vocational level compared with young people living in German-speaking cantons. Indeed, these latter are more likely to reach only a vocational level of education.

Table 4 shows that the parental background is more relevant than the individual variables of gender or birthplace in influencing the educational level attained by the descendants of migrants, particularly at tertiary level.^7^ The table also shows that parental background influences the type of education achieved by the migrants’ descendants, especially those parents with low SES. This means that the ‘ethno-class’ background has an impact especially on the less-privileged (those of Portuguese, Turkish, and ex-Yugoslavian origin) and less on those with more-heterogeneous SES profiles (here, those of Italian and Spanish origin). It confirms what other studies observed for former waves of second-generation migrants (Bolzman et al., 2003a, b): they attained a better education than migrants’ descendants who arrived more recently in Switzerland. In addition, the cantonal difference operationalised as language regions is particularly relevant when German- and French-speaking cantons are compared, confirming the prevalence of the apprenticeship system in German-speaking regions, while the young in French-speaking regions seem to have a greater likelihood of achieving tertiary studies. Nevertheless, it seems that the late tracking system in the French-speaking regions can also be a factor leading young people to be more likely to drop out early from the education system, as well as to remain with incomplete secondary studies.

## Discussion and Conclusions

Our results show an important relationship between national origin and parental SES. This picture of migration during the 1980s is conditioned by the labour and migration policies in Switzerland, which shaped a migration characterised by ‘ethno-classes’, relating the nationality and cultural capital of migrants. Migrants with low levels of educational capital who only have access to the lowest positions on the labour market were recruited in poorer countries than Switzerland, whilst those with higher levels of education—and coming from countries with similar or even higher levels of economic and social development as Switzerland—had the possibility to access the same positions as the native Swiss on the labour market and in social stratification. This relation between parents’ SES and nationality shaped by the Swiss labour-market and migration policy explains the existence of segmented assimilation among primo migrants—which may also occur in several European countries.

This congruency between primo migrants’ SES and nationality contributed to reinforcing the stigmatisation and discrimination of migrants from certain origins (Fibbi et al., (2022). In addition, it complicates the deconstruction of the ‘methodological nationalism’ in migration studies when studying education and occupation (Chimienti et al., 2021b; Wimmer & Glick Schiller, 2002). There are, however, exceptions to the rule: a percentage of ex-Yugoslavian and Portuguese nationals had a high SES in the ‘host country’ and occupy medium to high positions on the Swiss labour market (included in the type *Nationals and skilled migrants/mid to high SES*). In addition, the obtained typology also shows the changes in migrant profiles since the late 1980s, tending towards a more-skilled migration coming from neighbouring countries (Wanner & Steiner, 2018).

This equivalence between parents’ SES and nationality made us assume a stronger influence of parental background and socio-economic reproduction on the second generation. Our results indeed also show a segmented assimilation for migrants’ descendants in Switzerland. Whilst our results posit that the parental background is more relevant than the young people’s individual characteristics (birthplace and gender) in determining their educational outcomes, our results did not evidence a linear SES reproduction. The parental background mainly has an influence on the young people’s education when the parents have a low SES. The young still achieve a higher educational level than that achieved by their parents, as is the case for all second-generation respondents in our sample. Nevertheless, this social mobility is limited as, for migrants’ descendants with low parental SES, it mainly relies on vocational levels of education. This mobility is facilitated by the Swiss vocational training system, which offers access to study for a degree to those who did not want to choose or were unable to access a tertiary level of education and eases access to the labour market. This degree (Federal Certificate of Competence) even allows access to higher education in cases of reorientation, as highlighted by Gomensoro and Bolzman (2016). It represents a standard path for migrants’ descendants, given the highly selective (and discriminatory) characteristics of the Swiss education system, particularly in German-speaking cantons; it offers those from disadvantaged backgrounds the possibility to achieve a minimum qualification that is recognised on the labour market. It seems, however, that these young people and their migrant families do not have the (material, temporal or socio-cultural) resources (Triventi et al., 2021) they need to invest in reaching a higher educational level and overcoming the structural barriers they face.

To conclude, therefore, our hypothesis about ‘path dependence’ can be confirmed only partially in the LCS sample because, although the results show that migrant parental background and higher levels of education among their descendants are related, this is not equally valid for those who undertook vocational training. This pathway represents a concrete means of social mobility for young people with disadvantaged backgrounds. As observed by Gomensoro and Bolzman (2016), young people with higher educational aspirations are often pushed by the Swiss education system to follow vocational training even if they preferred to go to university. Indeed, if the vocational path were a stepping-stone for intergenerational social mobility, it may also be a trap for others with higher educational aspirations who might be discouraged or prevented from realising them. In this sense, the effective inclusion and support of migrants’ descendants with disadvantaged backgrounds and high aspirations remain, even today, a challenge for the Swiss educational system. Therefore, whilst this system succeeds in allowing the vast majority of young people to obtain an upper-secondary-level diploma, it maintains inequalities between young people of different social and national backgrounds.

The results presented in this paper also highlight the importance of early socialisation related to educational outcomes. Thus, having been born abroad represents a disadvantage in the Swiss educational system that clearly influences early school dropout by migrants’ young descendants. The Swiss education system seems not to be sufficiently prepared to host children born abroad; its early selective track limits the possibility to catch up for those who arrived at a later stage of schooling. Gomensoro and Bolzman (2015) note that *circa* 60% of children who arrived in Switzerland after the age of 10 did not continue their education and were on the labour market 7 years after having finished compulsory school. This could be even more problematic for the children of refugees, who may have to face years of intermittence or removal from the school system because of violent conflicts in their home countries or time spent waiting in camps (Chimienti et al., 2019; Koehler & Schneider, 2019).

As mentioned above, a limitation of this research is that the sample is concentrated on particular nationalities. While this focus allowed us to produce evidence of the problematic relation between parental migrant background and the educational outcome of their children, these results can also be understood as supporting the ethnicising congruence between migration and poverty, as criticised by Horvath (2019). Our results show the difficulty to go beyond the debate opposing “class versus ethnicity” in explaining educational inequalities between (and within) descendants of migrants compared to those of natives, as class and ethnicity cannot be separated in this case. Nevertheless, our results highlight above all the importance of the SES background in the educational outcomes over the nationality, because the observed congruence in our data is also a product of historical and institutional conditions.

Finally, in contrast to the results from previous research on the second generation, the gender of migrants’ descendants does not appear, in our analysis, to be an important variable with which to predict the educational level attained. Our analysis did not show significant results on how the parental background might differently influence young women’s education compared to that of young men. Previous research observed that gender differences tend to be equalised by the school system (Bloch & Hirsch, 2017; Chimienti et al., 2019; Farris & de Jong, 2013; Gomensoro & Bolzman, 2016). However, other studies have also shown that gender differences reappeared on entry to the labour market (Bolzman et al., 2003a; Farris & de Jong, 2013) as did ethnic and racial discrimination (Ossipow et al., 2019). Our lack of results about gender does not mean that these differences do not exist but simply that they need more-specific and in-depth research.

## Data Availability

The datasets analysed during the current study are available in the repository https://www.swissubase.ch.

## Footnotes

1 Dario Spini, Robin Tillmann, Felix Bühlmann, Nora Dasoki, Davide Morselli: LIVES FORS Cohort Survey Waves 1–7 [Dataset]. Swiss National Centre of Competence in Research LIVES – NCCR LIVES; Swiss Centre of Expertise in the Social Sciences – FORS. Financed by the Swiss National Science Foundation. Distributed by FORS, Lausanne, 2021. https://doi.org/10.23662/FORS-DS-937-5.

2 This degree is called a Federal Certificate of Competence. Certificat Fédéral de Compétences (CFC)/Eidgenössisches Fähigkeitszeugnis (EFZ).

3 According to the State Secretariat for Migration (SEM), in December 2021, the population from Ex-Yugoslavia and Turkey represented 12% and 3% of the resident population in Switzerland (Secrétariat d’État aux migrations, 2022).

4 Wanner and Steiner (2018) highlight that some differences were found among Southern-European migrants in Switzerland between 1980 and 1990. Portuguese migration was more clearly related to labour migration, whilst Italians and Spanish migrants showed more heterogeneous socio-cultural profiles.

5 The highest level of education stated as completed by the interviewee in the last interview.

6 The ESeC allows us to consider independent workers (for instance, a taxi driver) as being in a better position than an unskilled employee, although both may have the same level of education. Neither does it presume a person’s class, either according to their economic and cultural capital—as does Bourdieusian class analysis (opposing the dominant class, the middle class or *petite bourgeoisie* and the working or popular classes)—or in relation to a means of production, as does the Wright classification (Tillmann, 2020). Finally, the ESeC has a good fit with Swiss employment classifications.

7 In our model, gender is not significant, and birthplace is significant only regarding compulsory education (see Table 4).
